# Uncovering *Fibrocapsa japonica* (Raphidophyceae) in South America: First Taxonomic and Toxicological Insights from Argentinean Coastal Waters

**DOI:** 10.3390/toxins17080386

**Published:** 2025-07-31

**Authors:** Delfina Aguiar Juárez, Inés Sunesen, Ana Flores-Leñero, Luis Norambuena, Bernd Krock, Gonzalo Fuenzalida, Jorge I. Mardones

**Affiliations:** 1 División Ficología Dr. Sebastián Guarrera, Facultad de Ciencias Naturales y Museo (FCNyM), Universidad Nacional de La Plata (UNLP), Paseo del Bosque s/n, 1900 La Plata, Argentina; isunesen@fcnym.unlp.edu.ar; 2 Consejo Nacional de Investigaciones Científicas y Técnicas (CONICET), Godoy Cruz 2290, Ciudad Autónoma de Buenos Aires C1425FQB, Argentina; 3 Scottish Association for Marine Science, Scottish Marine Institute, Argyll, Oban PA37 1QA, UK; ana.floreslenero@sams.ac.uk; 4 Centro de Estudios de Algas Nocivas (CREAN), Instituto de Fomento Pesquero (IFOP), Puerto Montt 5501679, Chile; luis.norambuena@ifop.cl; 5 Alfred Wegener Institut-Helmholtz Zentrum für Polar- und Meeresforschung (AWI), Bremerhaven 27570, Germany; bernd.krock@awi.de; 6 Departamento de Ciencias Básicas, Facultad de Ciencias, Universidad Santo Tomas, Av. Ramon Picarte 1160, Valdivia 5090000, Chile; gfuenzalida2@santotomas.cl; 7 Centro de Investigación en Recursos Naturales y Sustentabilidad (CIRENYS), Universidad Bernardo OߣHiggins, Santiago 8370993, Chile

**Keywords:** harmful algal blooms (HABs), raphidophyte, cytotoxicity, phylogeny, photosynthetic pigments, Argentina

## Abstract

*Fibrocapsa japonica* (Raphidophyceae) is a cosmopolitan species frequently associated with harmful algal blooms (HABs) and fish mortality events, representing a potential threat to aquaculture and coastal ecosystems. This study provides the first comprehensive morphological, phylogenetic, pigmentary, and toxicological characterization of *F. japonica* strains isolated from Argentina. Light and transmission electron microscopy confirmed key diagnostic features of the species, including anterior flagella and the conspicuous group of mucocyst in the posterior region. Phylogenetic analysis based on the LSU rDNA D1–D2 region revealed monophyletic relationships with strains from geographically distant regions. Pigment analysis by HPLC identified chlorophyll-a (62.3 pg cell^−1^) and fucoxanthin (38.4 pg cell^−1^) as the main dominant pigments. Cytotoxicity assays using RTgill-W1 cells exposed for 2 h to culture supernatants and intracellular extracts showed strain-specific effects. The most toxic strain (LPCc049) reduced gill cell viability down to 53% in the supernatant exposure, while LC_50_ values ranged from 1.6 × 10^4^ to 4.7 × 10^5^ cells mL^−1^, depending directly on the strain and treatment type. No brevetoxins (PbTx-1, -2, -3, -6, -7, -8, -9, -10, BTX-B1 and BTX-B2) were detected by LC–MS/MS, suggesting that the cytotoxicity may be linked to the production of reactive oxygen species (ROS), polyunsaturated fatty acids (PUFAs), or hemolytic compounds, as previously hypothesized in the literature. These findings offer novel insights into the toxic potential of *F. japonica* in South America and underscore the need for further research to elucidate the mechanisms underlying its ichthyotoxic effect.

## 1. Introduction

The Class Raphidophyceae Chadefaud ex Silva comprises a small group of flagellated microalgae distributed across both freshwater and marine environments worldwide [1]. Among marine members, several species are recognized for their role in harmful algal blooms (HABs), which can severely impact aquatic ecosystems and economic activities [2]. Within this class, the genus *Fibrocapsa*, with *F. japonica* Toriumi & Takano as its only described species, is characterized by naked cells, two unequal flagella, numerous yellow-brown chloroplasts, and a cluster of naviculoid mucocysts located at the posterior end of the cell [3]. Despite its cellular fragility, which hinders preservation using conventional fixatives [4,5], *F. japonica* can be identified live using light microscopy based on its morphology and mucocyst organization [5,6].

Molecular studies using the internal transcribed spacer (ITS) region have shown that *F. japonica* forms a genetically diverse cosmopolitan population with high genetic polymorphism, possibly due to hybridization between parental haplotypes originating from isolated populations and dispersed via ballast water [7]. However, subsequent phylogenetic analyses have revealed the presence of distinct clades, including those of Italian strains [4,8], suggesting the existence of cryptic species. Similarly, large subunit ribosomal DNA (LSU rDNA) analyses have demonstrated that Brazilian strains cluster with those from Japan, Australia, and Germany, but diverge from the Italian lineage, further highlighting the species’ intraspecific genetic complexity [6].

*Fibrocapsa japonica* is both eurythermal and euryhaline, tolerating a broad range of temperatures and salinities [9,10]. This physiological plasticity may explain its widespread distribution, with reports spanning temperate and subtropical regions, including the Northwest Atlantic [11], the Mediterranean and Northern Adriatic Seas [8,12], the North Sea [13], the Southwestern [14,15], Eastern [16], and Northeastern Pacific [17], as well as the Western Pacific [18,19]. In South America, *F. japonica* has been recorded along the Southwest Atlantic coast between 22° S and 38° S, encompassing Brazilian, Uruguayan, and Argentinean waters [6,20,21,22].

Blooms of *F. japonica* have been implicated in mass fish mortalities, particularly in Japan, causing major economic losses [18,23]. While the exact mechanisms of ichthyotoxicity remain unclear, multiple hypotheses have been proposed. Initially, it was suggested that mucocyst discharge might block gill function, leading to hypoxia or asphyxia [10,24]. However, de Boer et al. [25] dismissed this hypothesis through experiments on common sole (*Solea solea*) larvae, which lack fully developed gills. Instead, toxicity may involve the production of brevetoxin-like compounds [26,27], reactive oxygen species (ROS) [28,29] and polyunsaturated fatty acids (PUFAs) [24,30]. The synergistic interaction between PUFAs and light-dependent hemolytic compounds has also been suggested as a key toxicological pathway [31]. Based on in vitro assays using RTgill-W1 fish gill cell lines, a widely used model for assessing algal cytotoxicity, Dorantes-Aranda et al. [32] proposed that lipid peroxidation driven by the synergistic action of ROS and PUFAs may contribute to cytotoxicity by generating compounds that induce physiological alterations in epithelial membranes, causing tissue damage. However, there is still debate as to which of these compounds are primarily responsible for the observed harmful effects.

**Figure 1:**
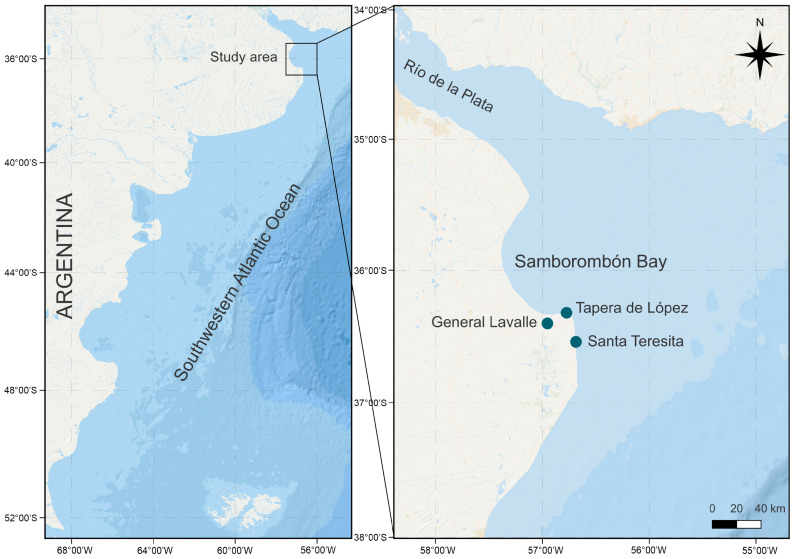
Location of the study area showing sampling sites.

HABs are increasingly recognized as a threat to marine biodiversity, human health, and coastal economies. Despite this growing concern, knowledge of raphidophyte blooms in Argentina remains limited [33]. Although *F. japonica* is known to occur in the region, most studies have focused on morphological and phylogenetic aspects, and no research has characterized its toxic potential in South America. Since 2008, our research group has led a long-term monitoring program in coastal marine waters of Buenos Aires Province, including the southern end of the Río de la Plata estuary (Samborombón Bay, Figure 1), a region of ecological and economic significance that supports artisanal fisheries and functions as a critical nursery area for commercially important species such as the whitemouth croaker (*Micropogonias furnieri*) [34]. Given the estuary’s role in sustaining biodiversity and fisheries, potential blooms of *F. japonica* could represent a significant environmental and socio-economic risk, highlighting the need to characterize its biology and toxicity.

This study aims to provide the first comprehensive taxonomic and toxicological characterization of *F. japonica* from Argentinean waters, integrating analyses of morphology, molecular markers, pigment profile, and cytotoxic potential. These findings provide novel insights into the diversity and harmful potential of this species in South America.

## 2. Results

### 2.1. Morphological Analysis

**Figure 2:**
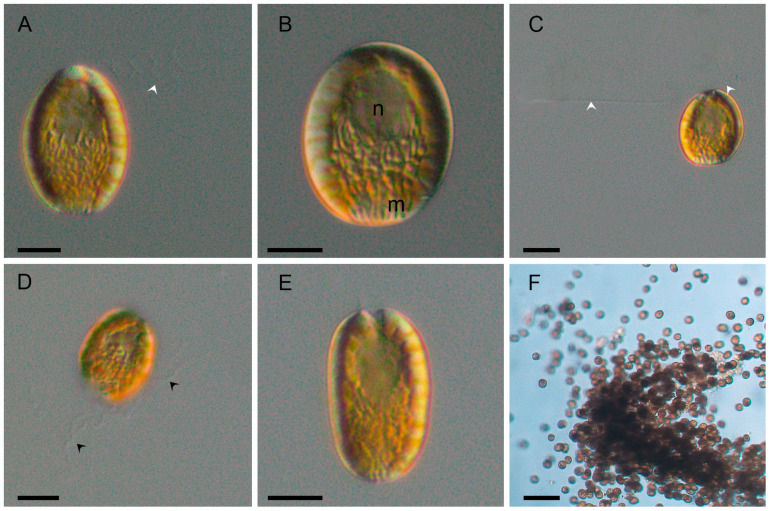
*Fibrocapsa japonica* cells observed by light microscopy (LM). (**A**) Cell of strain LPCc048 during exponential growth phase showing the anterior flagellum (white arrow). (**B**) Cell of strain LPCc048 showing the nucleus (n) and mucocysts (m). (**C**) Cell of strain LPCc050 during exponential growth phase showing two flagella (white arrow). (**D**) Cell of strain LPCc050 in exponential phase with discharged mucocysts (black arrows). (**E**) Cell of strain LPCc050 in stationary phase. (**F**) Strain LPCc048 aggregations. Scale bars = 10 µm (**A**–**E**); 50 µm (**F**).

The cells were brown-yellowish and oval to elliptical in shape (Figure 2A,B). During the stationary phase, some cells displayed rounded or subrectangular morphologies (Figure 2E). From all strains analyzed, cell length ranged from 19.8 to 36.1 µm (mean 27.4 ± 0.8 µm), width from 15.5 to 29.1 µm (mean 19.4 ± 0.7 µm), and the length-to-width ratio from 1.0 to 1.7 (mean 1.4 ± 0.0 µm) (n = 51). During the exponential growth phase, cells tend to aggregate in mucus-bound chains (Figure 2F).

Cells were biflagellated, with one anterior flagellum showing an undulating movement during swimming (Figure 2A,C, arrows), and a second flagellum directed posteriorly (Figure 2C, arrows). Both flagella emerged from a depression located in the anterior part of the cell (Figure 2A,C).

The posterior region of the cell contained numerous naviculoid mucocysts (Figure 2B or 2E). The nucleus was located centrally within the endoplasm (Figure 2B). Mucocysts discharged long mucilaginous threads outside the cell (Figure 2D, black arrows).

**Figure 3:**
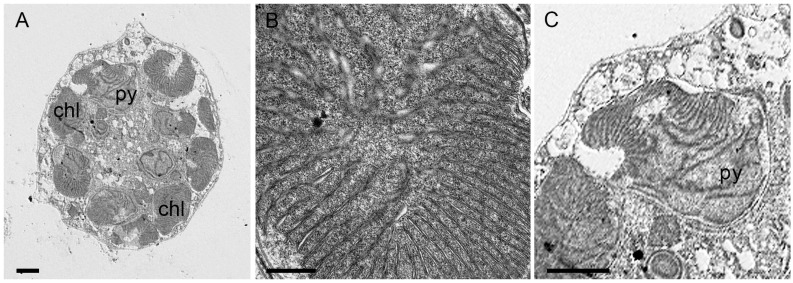
*Fibrocapsa japonica* cells under transmission electron microscopy (TEM). (**A**) Longitudinal section of an entire cell showing peripheral chloroplasts (chl) and chloroplast with a pyrenoid (py). (**B**) Detail of a chloroplast, note the bithylakoid lamellae. (**C**) Chloroplast with a pyrenoid (py) located anteriorly and oriented toward the cell center. Scale bars = 2 µm (**A**,**C**), 500 nm (**B**).

Chloroplasts were ellipsoidal to discoidal in shape, numerous and radially arranged in the ectoplasm (Figure 2A,E and Figure 3A, chl). Chloroplast lamellae were bithylakoidal (Figure 3B) and penetrated the pyrenoid (Figure 3C, py).

### 2.2. Phylogeny

**Figure 4:**
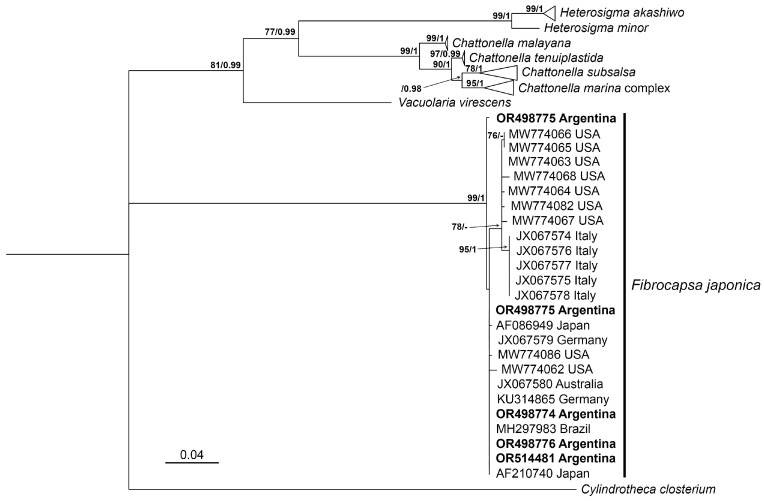
Maximum likelihood (ML) phylogenetic tree based on the large subunit ribosomal DNA (LSU rDNA) of Argentinean *F. japonica* sequences and related taxa. Sequences from Argentinean strains are highlighted in bold. Branch support values are indicated as ML/bootstrap and Bayesian inference (BI) posterior probabilities. Only support values greater than 60% for ML and 0.6 for BI are shown. The hyphen (-) indicates that the corresponding clade was not recovered in the BI analysis.

Phylogenetic analyses using Maximum Likelihood (ML) and Bayesian Inference (BI) produced congruent topologies, placing the sequences of the five Argentinean strains within the *Fibrocapsa japonica* clade, with strong statistical support (ML bootstrap = 99%; BI posterior probability = 1.0) (Figure 4).

The mean net genetic distance (*p*-distance) among all sequences within the clade was 0.01, while the *p*-distance among the Argentinean sequences ranged from 0.00000 to 0.007. Sequence OR498775, corresponding to strain LPCc050, exhibited the highest divergence within the Argentinean strains. In the ML tree, this sequence formed a sister lineage to the remaining *F. japonica* strains included in the dataset (Figure 4). Conversely, in the BI analysis, LPCc050 grouped with the sequence MW774086 from the United States, although with low posterior probability (<0.07).

### 2.3. Pigment Signature

High-performance liquid chromatography (HPLC) analysis of strain LPCc049 identified chlorophyll-a as the dominant pigment (62.3 pg cell^−1^), with chlorophyll-c2 (7.6 pg cell^−1^) and chlorophyll-c1 (4.8 pg cell^−1^) as accessory chlorophylls. Among carotenoids, fucoxanthin was the most abundant (38.4 pg cell^−1^), followed by violaxanthin (2.7 pg cell^−1^), antheraxanthin (1.1 pg cell^−1^), β-carotene (0.9 pg cell^−1^), and zeaxanthin (0.6 pg cell^−1^).

### 2.4. Cytotoxicity Assays

**Figure 5:**
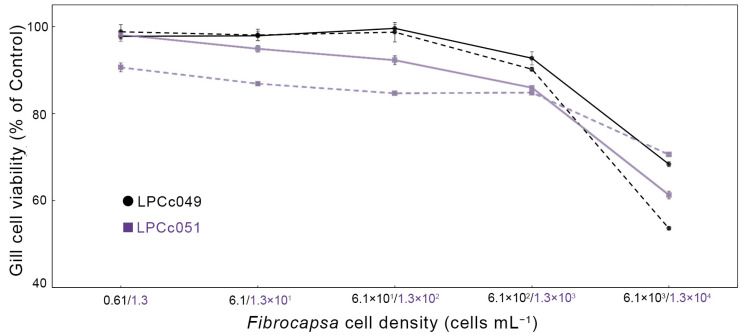
Cytotoxicity of intracellular (solid line) and supernatant (dotted line) treatments from strains LPCc049 (black) and LPCc051 (violet) against RTgill-W1 cell lines. Cell concentrations are color-coded to match each strain. Values represent means, and bars indicate standard error from four replicates.

Gill cell response after being exposed to the strains LPCc049 and LPCc051 for 2 h showed significant differences between treatments (intracellular and supernatant) and among tested cell concentrations (ANOVA, *p* < 0.01). For both strains, Tukey’s test indicated that the two highest cell concentrations (6.1 × 10^2^ and 6.1 × 10^3^ cells mL^−1^ for LPCc049, and 1.3 × 10^3^ and 1.3 × 10^4^ cells mL^−1^ for LPCc051) differed significantly from the lower concentrations in both treatments (*p* < 0.01). Strain LPCc049 was the most cytotoxic overall, with the supernatant treatment showing the strongest effect (ANOVA, *p* < 0.01), reducing viability down to 53%, while the intracellular compound lowered it to 68% at the highest concentration tested (Figure 5). In contrast, for strain LPCc051, the intracellular treatment caused the greatest reduction, decreasing cell survival down to 61%, while the supernatant had a milder effect, reducing viability to 70% (Figure 5).

Lethal concentration 50 (LC_50_) values for strain LPCc049 were 1.6 × 10^4^ cells mL^−1^ for the supernatant treatment and 1.9 × 10^6^ cells mL^−1^ for the intracellular extract. In contrast, LC_50_ values for LPCc051 were 1.3 × 10^5^ cells mL^−1^ and 4.7 × 10^5^ cells mL^−1^ for the supernatant and intracellular treatments, respectively. For the LPCc051 strain, although the intracellular treatment showed lower viability at the highest concentration tested, the supernatant consistently reduced viability at lower doses, resulting in a lower LC_50_ estimate.

### 2.5. Brevetoxin Analysis

LC–MS/MS analysis of the five *Fibrocapsa japonica* strains (LPCc048, LPCc049, LPCc050, LPCc058, and LPCc069) revealed no detectable levels of brevenal nor any of the screened brevetoxins (Table 1). Limits of detection (LoD) ranged between 0.03 and 0.06 pg cell^−1^, depending on strain and toxin analogue.

## 3. Discussion

### 3.1. Morphological Features and Taxonomic Identity

Observations using light and transmission electron microscopy revealed that Argentine strains closely match the type material originally described by Toriumi and Takano [23] later revised by Hara and Chihara [3], and further examined by Band-Schmidt et al. [5] and Branco et al. [6]. Notably, the presence of discoidal chloroplasts with a single pyrenoid, previously reported by Hara and Chihara [3], was also observed in our strains contrasting with the initial description by Toriumi and Takano [23]. Furthermore, Argentinean strains exhibited greater variability in cell size compared to those from Mexico and Brazil, with a broader range in length (19.8–36.1 µm) and width (15.5–29.1 µm) than Brazilian (21.8–23.6 µm length; 15.1–17.8 µm width) and Mexican strains (19.7–25.2 µm length; 15.7–18.4 µm width) [5,6]. This increased morphological variability may reflect intraspecific plasticity within Argentinean populations.

Culture-stage-dependent morphological changes, including cell rounding and aggregation during the stationary phase, were consistent with prior descriptions [3,6,9], reinforcing the taxonomic identification of our isolates as *F. japonica*.

### 3.2. Phylogenetic Relationships

Phylogenetic reconstruction based on the LSU rDNA D1–D2 region placed most Argentinean sequences within a well-supported *F. japonica* clade alongside strains from Japan, Australia, Germany, Brazil, and the United States. Interestingly, strain LPCc050 formed a sister linage, indicating potential divergence within the population. As reported previously [4,6,8], sequences from the Adriatic Sea clustered separately, forming a distinct subclade. The inclusion of U.S. sequences, not analyzed before, revealed a close relationship with the Italian subclade, challenging the hypothesis of Adriatic endemism. According to Klöpper et al. [4], the two major subclades of *F. japonica* may present morphological differences. Although LPCc050 did not cluster with the Adriatic or U.S. subclade, minor morphological differences were observed: LPCc050 exhibited a smaller average cell size (length: 24.9 ± 0.8 µm; width: 18.0 ± 0.6 µm) compared to the other Argentinean strains from the cosmopolitan clade (length: 28.0 ± 0.9 µm; width: 19.8 ± 0.8 µm) (Table 2). No other differences were detected under light microscopy. However, as mentioned in Section 3.1, some morphological variation was found when comparing Argentinean strains with other cosmopolitan strains. Given that different molecular markers (LSU vs. ITS) have been used across studies, future work integrating multiple loci and broader geographic representation will be essential to resolve the phylogeographic structure of this taxon.

### 3.3. Pigment Composition

The pigment composition of our *F. japonica* strain aligned with previous reports, with chlorophyll-a and fucoxanthin as the dominant pigments, accompanied by chlorophyll-c1/c2, β-carotene, violaxanthin, and zeaxanthin in lower proportions [5,16,35,36]. However, strain LPCc049 displayed a pigment profile that partially diverged earlier description. Pigments such as auroxanthin and fucoxanthinol, previously reported only in Japanese strains [35], were not detected and the presence of diadinoxanthin and antheraxanthin was inconsistent. These discrepancies likely reflect differences in culture conditions, physiological status, and analytical methodologies across studies. While this study focused on a single Argentinean strain, future analyses incorporating additional local isolates under standardized conditions would allow a more comprehensive characterization of the pigment profile of *F. japonica* population in the region.

### 3.4. Toxicity and Potential Harmfulness

This study provides the first evidence of cytotoxic potential in *F. japonica* strains isolated from South America. RTgill-W1 bioassays revealed significant intraspecific variation in toxicity, aligning with previous findings for this and other raphidophytes, such as *Chattonella marina* [32,33]. Among the two strains tested, LPCc049 exhibited the highest cytotoxicity, reducing cell viability down to 53% (supernatant) and 68% (intracellular extracts). Interestingly, for strain LPCc051, cell viability was lower in the intracellular extract than in the supernatant at the highest tested concentration. However, dose–response modeling revealed a lower LC_50_ for the supernatant treatment, emphasizing the importance of evaluating the entire concentration-response curve rather than relying on single data points.

Previous research has shown that *F. japonica* supernatants are toxic to *Artemia salina* larvae and capable of oxidizing hemoglobin in erythrocyte [36], due to reactive oxygen species (ROS). However, the ichthyotoxicity observed cannot be fully explained by ROS alone [32,37].

Strain-specific toxicity in *F. japonica* likely results from a complex interplay of environmental and physiological factors such as salinity, temperature, light intensity, and nutrient availability [36,38,39]. The growth phase is also critical determinant; for instance, de Boer et al. [25] reported increased toxicity in strain W420 during the late exponential phase. Additionally, methodological artifacts, such as cell rupture during centrifugation, may release intracellular compounds into the supernatant, potentially confounding the interpretation of extract-specific toxicity. Nonetheless, de Boer et al. [25] demonstrated that hemolytic compounds are released early in the growth cycle and at low cell concentrations, potentially providing *F. japonica* with a competitive ecological advantage. These variables help explain conflicting reports on whether extracellular or intracellular fractions exhibit greater toxicity. For example, Dorantes-Aranda et al. [32] observed higher intracellular toxicity in RTgill-W1 assays, although no significant differences were found between treatments. Similarly, Bridgers et al. [27] reported that both intracellular and extracellular extracts were toxic in fish bioassays using strains Fibro NZ and Fibro HH, with temporal and strain-specific variation.

Our LC–MS/MS analysis did not detect brevenal nor any of the screened brevetoxins in any Argentinean strain, corroborating previous studies [25,36,40]. This supports the hypothesis that the observed cytotoxicity may be attributed to other yet unidentified compounds [32]. Although not directly measured in this study, our findings align with previous suggestions that toxicity may be primarily driven by the synergistic effects of ROS and polyunsaturated fatty acids (PUFAs), such as eicosapentaenoic acid (EPA) and octadecatetraenoic acid (OTA) [25,31]. This contrasts with earlier reports of brevetoxins, which were based on ELISA assays [27] or chromatographic methods lacking mass spectrometry confirmation [26], both of which are prone to false positives or ambiguous identification.

Overall, our results highlight the need for further research aimed at identifying and characterizing the specific compounds responsible for *F. japonica* toxicity (particularly ROS, PUFAs, and hemolytic agents) in order to better understand their individual and combined contributions to strain-specific cytotoxicity.

### 3.5. Ecological and Monitoring Implications

*Fibrocapsa japonica* has been linked to severe fish kills in Japan, leading to substantial economic losses [18,23]. In the Southwest Atlantic, the species has been documented based solely on morphology in coastal waters of Argentina and Uruguay [21,22]. Although no fish mortalities have been attributed to *F. japonica* in the region to date, its ability to tolerate a wide range of environmental conditions, such as the variable temperatures (20.6–23.6 °C) and salinities (15.9–20.8) measured during strain isolation (Table 2), along with its capacity to produce resting cysts [41] and exhibit cytotoxicity highlight its potential ecological risk, particularly in sensitive estuarine systems like the Río de la Plata.

Given these attributes, we recommend that *F. japonica* be included as a target species in HAB monitoring programs in Argentina, especially in regions with commercially important fish stocks. Incorporating this species into early detection and response strategies could be crucial to mitigate potential impacts.

## 4. Materials and Methods

### 4.1. Isolation and Culture of Microalgae Strains

Strains of *Fibrocapsa japonica* were obtained from surface water samples collected with a 30 µm mesh phytoplankton net from three places between February 2017 and April 2021 (Table 2). Isolation was performed using a micropipette under a Zeiss Axiovert 40 CFL inverted microscope. The isolates were incubated first in filtered estuarine water (salinity of 20), corresponding to the salinity measured at the time of sampling. After initial growth, cultures were gradually transferred to filtered natural marine water (salinity of 30), enriched with Guillard’s f/2 medium (Sigma Aldrich, Saint Louis, MO, USA). Cultures were maintained at 16 °C under a 12:12 h light:dark cycle with cool-white fluorescent lighting, following the protocol of Sunesen et al. [42]. All cultures were kept in 250 mL sterile Erlenmeyer flasks under controlled conditions. All strains are maintained in the culture collection of the Herbarium of the División Ficología ‘Dr. Sebastián A. Guarrera’.

### 4.2. Microscopy

#### 4.2.1. Light Microscopy

Cells were observed alive using an Axiovert 40 CFL inverted microscope (Zeiss Microimaging, Goettingen, Germany) equipped with phase contrast and differential interference contrast (DIC) optics, along with an AxioCam 208c digital camera, and with a Leica DMLA microscope (Leica Microsystems, Wetzlar, Germany) fitted with DIC and a DFC420c digital camera.

#### 4.2.2. Transmission Electron Microscopy

Cells of the LPCc048 strain were initially fixed in 1% glutaraldehyde in culture medium for 1 h at room temperature. Subsequently, they were transferred to a solution of 2.5% glutaraldehyde in culture medium at 4 °C and left overnight. After centrifugation, cells were rinsed three times in 0.1 M Na-cacodylate buffer (pH 7.2) supplemented with culture medium for 10 min each rinse. Post-fixation was performed using 1% osmium tetroxide in the same buffer for 1 h at 4 °C, followed by three rinses with distilled water for 10 min each. Cells were then stained with 2% aqueous uranyl acetate solution for 1 h at room temperature under a hood, and samples were rinsed thrice with distilled water for 10 min each.

Dehydration was carried out through a graded ethanol series (30%, 50%, 70% and 95%) for 10–15 min per step, followed by two times in pure ethanol (30 min each) and by two incubations in absolute acetone (100%) for 15 min each. After dehydration, the cells were transferred into a 1:1 solution of 100% acetone and Epon 812 embedding resin (EMS, Hatfield, PA, USA) overnight. After 24 h, the mixture was replaced with pure resin, which was left in a vacuum oven at room temperature for 8 h. A final embedding step was performed in new resin, polymerized at 60 °C for 24 h. Ultrathin sections (70 nm) were obtained using a Leica EM UC7 ultramicrotome (Leica Microsystems, Wetzlar, Germany) fitted with a diamond knife. Sections were mounted on hexagonal copper grids (G200, EMS, Hatfield, PA, USA), and then stained with 2% uranyl acetate in methanol for 2 min, and lead citrate for 5 min. Imaging was performed on a Zeiss LIBRA 120 TEM (Carl Zeiss, Jena, Germany) operating at 120 kV.

### 4.3. DNA Extraction, Amplification, Sequencing and Phylogenetic Analysis

Genomic DNA was isolated from five *F. japonica* strains harvested during the exponential growth phase, using the Plan Genomic DNA Purification Kit (Thermo Fisher Scientific, Waltham, MA, USA) according to the manufacturer’s instructions. The D1–D2 region of the LSU rDNA gene was amplified using the primers D1R (ACCCGCTGAATTTAAGCATA) and D2C (CCTTGGTCCGTGTTTCAAGA) [43,44]. PCR amplification was performed using Platinum™ Taq DNA Polymerase (Thermo Fisher Scientific, Waltham, MA, USA), with an initial denaturation at 95 °C for 5 min, 40 cycles of 95 °C for 1 min, 55 °C for 1:20 min, and 72 °C for 2 min; followed by a final extension at 72 °C for 10 min. PCR products were verified by electrophoresis on 1.5% agarose gel and subsequently sent to Macrogen Sequencing Facility (Macrogen^®^, Seoul, South Korea). Sequence alignments (700 base pairs) were generated using ClustalX [45], incorporating reference sequences available in GenBank (Table S1).

Phylogenetic relationships were reconstructed using the maximum likelihood (ML) method under the Tamura–Nei model with a discrete Gamma distribution (TN93+G) [46], selected as the best-fitting model based on the lowest BIC and AICc scores using the MEGA X (version 10.2.6) software [47]. The estimated Gamma shape parameter was 0.75. No invariant sites were assumed in this model. Node support was assessed through 1000 bootstrap replicates. Net mean *p*-distances between the sequences were calculated without corrections for site-specific substitution saturation, transition/transversion biases, or rate variation among sites [46]. The analyses were performed using the same software. Additionally, Bayesian Inference (BI) analysis was conducted with MrBayes V3.2 [48], sampling across the entire general time reversible (GTR) model space with a gamma-distributed rate variation among sites. Two independent MCMC runs with four chains each were executed for 1,000,000 generations, sampling every 100 generations. The first 25% of trees were discarded as burn-in. Posterior probabilities were calculated from the remaining trees, and convergence was confirmed by split frequency values <0.02. The phylogenetic trees were rooted with a *Cylindrotheca closterium* (Ehrenberg) Reimann & J.C.Lewin sequence.

### 4.4. Pigment Analysis

An aliquot of 40 mL of the *Fibrocapsa japonica* LPCc049 culture, harvested in the exponential growth phase, was used for pigment analysis. The sample was centrifuged at 3000× *g* for 20 min, and the resulting pellet was extracted in 1.0 mL acetone (90%) after 60 s of probe sonication. Photosynthetic pigments were analyzed using a Shimadzu high-performance liquid chromatography (HPLC) system equipped with a Sil-10AF autosampler, LC-10AT quaternary pump, DGU-14A degasser, SPD-M20A diode array detector, and CBM-20A System Controller (Shimadzu Corporation, Kyoto, Japan). Chromatographic separation was performed using an ACE C18 PFP column (150 × 4.6 mm, 3 µm particle size; Advanced Chromatography Technologies, Aberdeen, UK) maintained at 40 °C. The mobile phases consisted of methanol:225 mM ammonium acetate (82:12 *v*/*v*) as phase A and ethanol as phase B. The gradient elution was programmed as follows: initial condition of 4% B, linearly increased to 38% B in 18 min, followed by a rapid increase to 57% B in 0.1 min, and then a linear gradient to 100% B in 18 min. The gradient returned to initial conditions in 0.1 min, and the total run time was 41 min.

Pigments were detected and quantified using a diode array detector scanning from 300 to 720 nm. Identification was based on the comparison of the retention times and spectral characteristic with certified pigment standards. These included alloxanthin, antheraxanthin, β-carotene, 19-butanoyloxyfucoxanthin, chlorophyll-a, chlorophyll b, chlorophyll-c1, chlorophyll-c2, chlorophyll-c3, 9-cis-neoxanthin, diatoxanthin, diadinoxanthin, dinoxanthin, fucoxanthin, gyroxanthin, 19-hexanoyloxyfucoxanthin, 19-hexanoyloxy-ketofucoxanthin, peridinin, prasinoxanthin, lutein, violaxanthin and zeaxanthin, all acquired from DHI (DHI Laboratory Products, Hoersholm, Denmark). Ammonium acetate, ethanol, and methanol used were of HPLC gradient grade (Merck, Darmstadt, Germany).

### 4.5. Toxicity

#### 4.5.1. Gill Cell Assay with Fibrocapsa Strains

The cell line RTgill-W1 [49] was sourced through the American Type Culture Collection (CRL-2523, ATCC, Manassas, VA, USA). Cells were cultured according to Dorantes-Aranda et al. [50] following the steps described by Flores-Leñero et al. [51]. Briefly, RTgill-W1 were cultured in 25 cm^2^ culture flasks using Leibovitz’s L-15 medium (L1518 Sigma, Aizu, Japan), enriched with 10% (*v*/*v*) fetal bovine serum (FBS, 12003C, Sigma) and an antibiotic-antimycotic mix (A5955, Sigma). Cultures were kept in the dark at 19 °C, with medium renewed every 48–72 h, and subculturing at 80–90% confluence using TrypLE™ Express (Gibco™).

Gill cell viability was assessed using the resazurin-based alamarBlue assay according to Dorantes-Aranda et al. [50] using the methodology described by Aguiar Juárez et al. [33]. Briefly, confluent cells were trypsinized, counted using a hemocytometer, and adjusted to 2 × 10^5^ cells mL^−1^. Cells were seeded in quadruplicate (n = 4 replicates per treatment) in 96-well flat-bottom microplates (3860-096, Iwaki, Shizuoka, Japan) with 100 µL per well. After a 48 h attachment period at 19 °C in darkness, PBS was used to rinse the cells after removing the L-15 medium. The RTgill-W1 cells were exposed to filter-sterilized (0.22 µm) intracellular and supernatant (extracellular) extracts using the LPCc049 and LPCc051 strains at cell densities of 6.1 × 10^3^, 6.1 × 10^2^, 6.1 × 10^1^, 6.1 and 0.61 cells mL^−1^, and 1.3 × 10^3^, 1.3 × 10^2^, 1.3 × 10^1^, 1.9 and 0.19 cells mL^−1^, respectively. Cultures were in the late exponential growth phase.

The supernatant treatment was prepared by diluting cultures to the desired cell abundances using seawater enriched with culture medium, centrifuging them at 3000 rpm for 10 min to pellet the cells, and filtering the supernatant through a 0.22 µm nylon membrane. For the intracellular treatment, the cell pellets were resuspended, sonicated for 2 min (amplitude of 10 µm peak to peak at 17 °C), and filtered using a nylon filter (0.22 µm). The exposure to the supernatant and intracellular compounds was carried out for 2 h at 19 °C in the dark. After exposure, viability was assessed using 5% alamarBlue (DAL1025, Invitrogen, Waltham, MA, USA) in L-15/ex medium after 2 h incubation in the dark. Fluorescence emitted was recorded with a microplate reader (FLUOstar Omega, BMG Labtech 415-2871), using excitation and emission filters at 540 and 590 nm, respectively. Gill cell viability was calculated as the percentage response of each treatment relative to the control (% of control).

#### 4.5.2. Brevetoxin Screening

For brevetoxin analysis, aliquots from exponentially growing cultures with previously quantified cell densities were centrifuged at 1000× *g* for 15 min. Pellets were placed into 1.5 mL Eppendorf tubes and kept at –20 °C until further analysis.

Samples were thawed at room temperature and subjected to cell lysis by sonication. Toxins were extracted using methanol, following standardized protocols for the recovery of lipophilic toxins. Ultra-high-performance liquid chromatography coupled to tandem mass spectrometry (UPLC–MS/MS) was employed to analyze the methanolic extracts, using a XEVO TQ-XS system (Waters, Eschborn, Germany) with an electrospray ionization (ESI) source operating in positive mode. Toxins were separated using reverse-phase chromatography on a C18 column (Purospher STAR RP-18 endcapped, 2 μm, Hibar HR, 50 × 2.1 mm, 1.7 μm; Merk, Darmstadt, Germany) at 40 °C. The mobile phase consisted of water with 0.01% formic acid and 0.05% ammonium hydroxide (phase A) and acetonitrile with 0.01% formic acid (phase B), with a flow rate of 0.6 mL min^−1^ and an initial isocratic elution with 5% phase B for 1.5 min followed by a linear gradient to 100% phase B for 2 min and 3 min isocratic elution with 100% phase B. After the chromatographic elution, the eluent composition was returned to initial conditions within 0.5 min. The injection volume was 0.5 μL.

Brevetoxins were detected using selected reaction monitoring (SRM) in the positive ion mode. The following MS parameters were applied: capillary voltage, 3.49 kV; source temperature, 150 °C; desolvation temperature, 600 °C; desolvation gas, 1000 L h^−1^; cone gas, 150 L h^−1^; nebulizer gas, 7.0 bar; collision gas flow, 0.15 mL min^−1^; dwell time, 0.01 s; and cone voltage, 20 V. The mass transitions used for the detection of brevetoxins and brevenal are listed in Table 1.

### 4.6. Statistical Analysis

To assess the effects of both cytotoxic response (cell viability) and microalgal concentration, a two-way analysis of variance (ANOVA) was conducted. Prior to ANOVA, assumptions of normality and homogeneity of variances were verified using the Shapiro–Wilk and Levene tests, respectively. Post hoc pairwise comparisons among treatments and concentrations were performed using Tukey’s Honestly Significant Difference (HSD) test to identify statistically significant differences. The median lethal concentration (LC_50_) values were estimated by fitting a log-logistic dose–response model with the drc package [52]. The most appropriate model was selected using the mselect function. LC_50_ estimates are reported with 95% confidence intervals. All statistical tests and model fittings were carried out in R software version 4.3.2 [53].

## Figures and Tables

**Table 1:** LC–MS/MS transitions (parent ion > daughter ion), applied collision energies, and names of the screened compounds.

***m*** **/*z* Parent Ion**	***m*** **/*z* Daughter Ion**	**Collision Energy [eV]**	**Compound Name**	
657.4	159.1	30	Brevenal	
657.4	255.2	30		
657.4	603.4	30		
657.4	621.4	30		
657.4	639.4	30		
867.1	385.2	15	PbTx-1	
867.1	849.2	15		
895.5	455.2	25	PbTx-2	
895.5	877.2	15		
897.5	725.2	25	PbTx-3	
897.5	879.5	15		
911.5	875.1	25	PbTx-6	
911.5	893.2	14		
869.5	851.5	30	PbTx-7	
917.9	899.9	30	PbTx-8	
899.5	881.5	25	PbTx-9	
911.5	875.1	15		
871.5	853.5	30	PbTx-10	
985.5	967.5	30	BTX-B1	
1034.6	753.0	30	BTX-B2	
1034.6	929.0	30		
1034.6	1016.6	30		

**Table 2:** Isolation data and average of cell size of the five monoclonal strains of *Fibrocapsa* from Argentinean coastal waters.

**Strain**	**Sample Site and Herbarium Code**	**Water Temperature and Salinity**	**Date**	**Isolator**	**GenBank Accession Number**	**Cell Length (µm)**	**Cell Width (µm)**
LPCc058	Santa Teresita LPC 11471	23.0 °C 20.7	2 February 2017	Sunesen I.	OR514481	23.4 ± 0.7	16.6 ± 0.9
LPCc048	Tapera de López LPC 13715	21.5 °C 18.9	15 March 2020	Aguiar Juárez D.	OR498773	30.8 ± 1.2	22.4 ± 1.1
LPCc049	Tapera de López LPC 13727	23.6 °C 20.0	28 February 2021	Aguiar Juárez D.	OR498774	27.5 ± 0.8	22.1 ± 1.0
LPCc050	General Lavalle LPC 13732	20.6 °C 15.9	29 March 2021	Aguiar Juárez D.	OR498775	24.9 ± 0.8	18.0 ± 0.6
LPCc051	Tapera de López LPC 13734	23.0 °C 20.8	18 April 2021	Aguiar Juárez D.	OR498776	30.3 ± 0.9	17.9 ± 0.3

## Data Availability

The original contributions presented in this study are included in the article/supplementary material. Further inquiries can be directed to the corresponding authors.

## Supplementary Materials

The following supporting information can be downloaded at https://www.mdpi.com/article/10.3390/toxins17080386/s1, Table S1: List of species, countries, strains codes, Genbank accession number and references used in LSU rDNA phylogeny [54,55,56,57,58,59,60,61,62].
