# Supplementary material for: Uncovering Fibrocapsa japonica (Raphidophyceae) in South America: First Taxonomic and Toxicological Insights from Argentinean Coastal Waters
Source: Toxins (Basel). 2025 Jul 31;17(8):386. doi: 10.3390/toxins17080386 (PMC12390448; doi:10.3390/toxins17080386)
Supplement: Supplementary file 1 [file toxins-17-00386-s001.zip › toxins-3722754-supplementary.pdf]

Supplementary materials: Uncovering Fibrocapsa *japonica* (Raphidophyceae) in South America: First Taxonomic and Toxicological Insights from Argentinean Coastal Waters

Delfina Aguiar Juárez, Inés Sunesen, Ana Flores-Leñero, Luis Norambuena, Bernd Krock, Gonzalo Fuenzalida and Jorge I. Mardones

Table S1. List of species, countries, strains codes, Genbank accession number and references used in LSU rDNA phylogeny.

Species	Country	Strain	Access Number	References
Chattonella antiqua	Japan	CCMP2052	FJ030891	Riisberg et al. [54]
Chattonella malayana	Malaysia	CtBk01	LC721667	Lum et al. [55]
Chattonella malayana	Malaysia	CtBk02	LC721668	Lum et al. [55]
Chattonella malayana	Malaysia	CtBk03	LC721669	Lum et al. [55]
Chattonella marina	Argentina	LPCc045	OR096705	Aguiar Juárez et al. [33]
Chattonella marina	Argentina	LPCc046	OR088598	Aguiar Juárez et al. [33]
Chattonella marina	Argentina	LPCc047	OR095727	Aguiar Juárez et al. [33]
Chattonella marina	Russia	VL508	LC633841	Lum et al. [56]
Chattonella marina var. *antiqua*	Brazil	CASC1	MH297980	Branco et al. [6]
Chattonella marina var. *antiqua*	Japan	NIES1	AB217631	Unpublished
Chattonella marina var. *antiqua*	Japan	NIES86	AB217868	Unpublished
Chattonella marina var. *antiqua*	NZ	CAWR23	MW177907	Unpublished
Chattonella marina var. *ovata*	Japan	NIES603	AB217640	Unpublished
Chattonella ovata	Hong Kong	Nd	AY704163	Kai et al. (2006)
Chattonella subsalsa	Brazil	Ca11	MH297976	Branco et al. [6]
Chattonella subsalsa	Brazil	CSRJ1	MH297979	Branco et al. [6]
Chattonella subsalsa	Iran	CHPI36	JF896100	Attaran-Fariman & Bolch [57]
Chattonella subsalsa	Italy	CRIM_H	JX067561	Klöpper et al. [4]
Chattonella subsalsa	USA	CS0707-1	KP702884	Engesmo et al. [58]
Chattonella tenuiplastida	Malaysia	CtaLuT01	LC721670	Lum et al. [55]
Chattonella tenuiplastida	Malaysia	St1409S2	LC721671	Lum et al. [55]
Chattonella tenuiplastida	Malaysia	LChTD267F2	LC721672	Lum et al. [55]
Fibrocapsa japonica	Argentina	LPCc058	OR514481	**This work**
Fibrocapsa japonica	Argentina	LPCc048	OR498773	**This work**
Fibrocapsa japonica	Argentina	LPCc049	OR498774	**This work**
Fibrocapsa japonica	Argentina	LPCc050	OR498775	**This work**
Fibrocapsa japonica	Argentina	LPCc051	OR498776	**This work**
Fibrocapsa japonica	Germany	Fibjap_JG	JX067579	Klöpper et al. [4]
Fibrocapsa japonica	Germany	SCCAP K-0542	KU314865	Unpublished
Fibrocapsa japonica	Australia	CCMP1661	JX067580	Klöpper et al. [4]
Fibrocapsa japonica	Brazil	FJSC3	MH297983	Branco et al. [6]
Fibrocapsa japonica	USA	NCCW-ARC10	MW774062	Unpublished
Fibrocapsa japonica	USA	UNCW-ARC11	MW774063	Unpublished
Fibrocapsa japonica	USA	UNCW-ARC12	MW774064	Unpublished
Fibrocapsa japonica	USA	UNCW-ARC13	MW774065	Unpublished
Fibrocapsa japonica	USA	UNCW-ARC15	MW774066	Unpublished
Fibrocapsa japonica	USA	UNCW-ARC16	MW774067	Unpublished
Fibrocapsa japonica	USA	UNCW-ARC17	MW774068	Unpublished
Fibrocapsa japonica	USA	UNCW-ARC40	MW774082	Unpublished
Fibrocapsa japonica	USA	UNCW-ARC53	MW774086	Unpublished
Fibrocapsa japonica	Italy	FRIM_C	JX067574	Klöpper et al. [4]

Fibrocapsa japonica	Italy	FRIM_D	JX067575	Klöpper et al. [4]
Fibrocapsa japonica	Italy	FRIM_E	JX067576	Klöpper et al. [4]
Fibrocapsa japonica	Italy	FRIM_A	JX067577	Klöpper et al. [4]
Fibrocapsa japonica	Italy	FRIM_B	JX067578	Klöpper et al. [4]
Fibrocapsa japonica	Japan	CAWR03	AF210740	Tyrrell et al. [59]
Fibrocapsa japonica	Japan	LB 2162	AF086949	Unpublished
Heterosigma akashiwo	Argentina	LPCc052	OR494634	Aguiar Juarez [60]
Heterosigma akashiwo	Argentina	LPCc053	OR494633	Aguiar Juarez [60]
Heterosigma akashiwo	Argentina	LPCc054	OR494680	Aguiar Juarez [60]
Heterosigma akashiwo	Argentina	LPCc055	OR494681	Aguiar Juarez [60]
Heterosigma akashiwo	Argentina	LPCc056	OR494693	Aguiar Juarez [60]
Heterosigma akashiwo	Argentina	LPCc057	OR514480	Aguiar Juarez [60]
Heterosigma akashiwo	Argentina	LPCc031	OR514479	Aguiar Juarez [60]
Heterosigma akashiwo	Argentina	LPCc042	PP799058	Aguiar Juarez [60]
Heterosigma akashiwo	Australia	CAWR04	KP702890	Engesmo et al. [58]
Heterosigma akashiwo	Brazil	Ha1	MH297981	Branco et al. [6]
Heterosigma akashiwo	Canada	UBC-B	DQ470659	Ki & Han [61]
Heterosigma akashiwo	Chile	HA_CREAN	OL764492	Flores-Leñero et al. [51]
Heterosigma akashiwo	USA	CCMP2274	JX067555	Klöpper et al. [4]
Heterosigma akashiwo	Korea	HAKS-01	DQ470660	Ki & Han [61]
Heterosigma akashiwo	NZ	CAWR13	KP702895	Engesmo et al. [58]
Heterosigma minor	USA	HA0504-1	KP780268	Engesmo et al. [58]
Vacuolaria virescens	Nd	Nd	AF409125	Ben Ali et al. [62]

4. Klöpper, S.; John, U.; Cembella, A.D. A new Mediterranean genotype of *Fibrocapsa* sp. In Proceedings of the 12th International Conference on Harmful Algae, Copenhagen, Denmark, 4–8 September 2006 ; Moestrup, Ø., Eds.; International Society for the Study of Harmful Algae; Intergovernmental Oceanographic Commission of UNESCO: Copenhagen, Denmark, 2008; pp. 259–261.

6. Branco, S.; Almeida, L.L.; Alves-de-Souza, C.; Oliveira, M.M.M.; Proença, L.A.O.; Menezes, M. Morphological and genetic characterization of bloom-forming raphidophyceae from Brazilian coast. *Phycol. Res*. **2019**, 67, 279–290 . https://doi.org/10.1111/pre.12377.

33. Aguiar Juárez, D.; Mardones, J.I.; Flores-Leñero, A.; Norambuena, L.; Paredes-Mella, J.; Sar, E.A.; Sunesen, I. First description of the fish-killing raphidophyceae *Chattonella marina* complex in Argentina: From genetics to ichthyotoxicity unveiled. *Harmful Algae* **2025**, 142, 102804. https://doi.org/10.1016/j.hal.2025.102804.

51. Flores-Leñero, A.; Vargas-Torres, V.; Paredes-Mella, J.; Norambuena, L.; Fuenzalida, G.; Lee-Chang, K.; Mardones, J.I. *Heterosigma akashiwo* in Patagonian fjords: Genetics, growth, pigment signature and role of PUFA and ROS in ichthyotoxicity. *Toxins* **2022**, 14, 577. https://doi.org/10.3390/toxins14090577.

54. Riisberg, I.; Orr, R.J.S.; Kluge, R.; Schalchian-Tabrizi, K.; Bowers, H.A.; Patil, V.; Edvardsen, B.; Jakobsen, K.S. Seven gene phylogeny of Heterokonts. *Protist* **2009**, 160, 191–204. https://doi.org/10.1016/j.protis.2008.11.004.

55. Lum, W.M.; Lim, H.C.; Lau, W.L.S.; Law, I.K.; Teng, S.T.; Benico, G.; Leong, S.C.Y.; Takahashi, K.; Gu, H.; Lirdwitayaprasit, T.; et al. Description of two new species *Chattonella tenuiplastida* sp. nov. and *Chattonella malayana* sp. nov. (Raphidophyceae) from South China Sea, with a report of wild fish mortality. *Harmful Algae* **2022**, 118, 102322. https://doi.org/10.1016/j.hal.2022.102322.

56. Lum, W.M.; Benico, G.; Doan-Nhu, H.; Furio, E.; Leaw, C.P.; Leong, S.C.Y.; Lim, P.T.; Lim, W.A.; Lirdwitayaprasit, T.; Lu, S.; et al. The harmful raphidophyte *Chattonella* (Raphidophyceae) in Western Pacific: Its red tides and associated fisheries damage over the past 50 years (1969–2019). *Harmful Algae* **2021**, 107, 102070. https://doi.org/10.1016/j.hal.2021.102070.

57. Attaran-Fariman, G.; Bolch, C.J.S. Morphology and genetic affinities of a novel *Chattonella* isolate (Raphidophyceae) isolated from Iran's South Coast (Oman Sea). *Turk. J. Botany* **2014**, 38, 156–168.
58. Engesmo, A.; Eikrem, W.; Seoane, S.; Smith, K.; Edvardsen, B.; Hofgaard, A.; Tomas, C.R. New insights into the morphology and phylogeny of *Heterosigma akashiwo* (Raphidophyceae), with the description of *Heterosigma minor* sp. nov. *Phycologia* **2016**, 55, 279–294. https://doi.org/10.2216/15-115.1.
59. Tyrrell, J.V.; Bergquist, P.R.; Bergquist, P.L.; Scholin, C.A. Detection and enumeration of Heterosigma akashiwo and *Fibrocapsa japonica* (Raphidophyceae) using rRNA-targeted oligonucleotide probes. *Phycologia* **2001**, 40, 457–467. https://doi.org/10.2216/i0031-8884-40-5-457.1.
60. Aguiar Juárez, D. Dinámica espacio-temporal del fitoplancton de la Bahía Samborombón y cracterización integral de especies ictiotóxicas. Ph.D. Thesis, Universidad Nacional de La Plata, La Plata, Argentina, 2025.
61. Ki, J.S.; Han, M.S. Nuclear rDNA and chloroplast rbcL, rbcS and IGS sequence data, and their implications from the Japanese, Korean, and North American harmful algae, *Heterosigma akashiwo* (Raphidophyceae). *Environ. Res*. **2007**, 103, 299–304. https://doi.org/10.1016/j.envres.2006.08.014.
62. Ben Ali, A.; De Baere, R.; De Wachter, R.; Van De Peer, Y. Evolutionary relationships among heterokont algae (the autotrophic Stramenopiles) based on combined analyses of small and large subunit ribosomal RNA. *Protist* **2002**, 153, 123–132. https://doi.org/10.1078/1434-4610-00091.
